# Power Spectral Density and Default Mode Network Connectivity in Generalized Epilepsy Syndromes: What to Expect from Drug-Resistant Patients

**DOI:** 10.3390/biomedicines12122756

**Published:** 2024-12-02

**Authors:** Cătălina Elena Bistriceanu, Georgiana-Anca Vulpoi, Alin Ciubotaru, Iulian Stoleriu, Dan Iulian Cuciureanu

**Affiliations:** 1 Neurology Department, Faculty of Medicine, University of Medicine and Pharmacy “Grigore T. Popa”, 16 Universitatii Street, 700115 Iasi, Romania; vulpoi.anca@yahoo.com (G.-A.V.); alinciubotaru94@yahoo.com (A.C.); cuciureanudan@yahoo.com (D.I.C.); 2 Elytis Hospital Hope, 43A Gheorghe Saulescu Street, 700010 Iasi, Romania; 3 Dorna Medical, 700022 Iasi, Romania; 4 Department of Neurology, Rehabilitation Hospital, 700661 Iasi, Romania; 5 Faculty of Mathematics, ”Alexandru Ioan Cuza” University, 11 Bd. Carol I, 700506 Iasi, Romania; stoleriu@yahoo.com; 6 Neurology Department I, “Prof. Dr. N. Oblu” Emergency Clinical Hospital, 2 Ateneului Street, 700309 Iasi, Romania

**Keywords:** idiopathic generalized epilepsy (IGE), default mode network (DMN), linear connectivity, power spectral density (PSD), drug-resistant IGE (DR-IGE)

## Abstract

**Background:** Recent studies have described unique aspects of default mode network connectivity in patients with idiopathic generalized epilepsy (IGE). A complete background in this field could be gained by combining this research with spectral analysis. **Objectives:** An important objective of this study was to compare linear connectivity and power spectral densities across different activity bands of patients with juvenile absence epilepsy (JAE), juvenile myoclonic epilepsy (JME), generalized tonic–clonic seizures alone (EGTCSA), and drug-resistant IGE (DR-IGE) with healthy, age-matched controls. **Methods:** This was an observational case–control study. We performed EEG spectral analysis in MATLAB and connectivity analysis with LORETA for 39 patients with IGE and 12 drug-resistant IGE (DR-IGE) and healthy, age-matched subjects. We defined regions of interest (ROIs) from the default mode network (DMN) and performed connectivity statistics using time-varying spectra for paired samples. Using the same EEG data, we compared mean power spectral density (PSD) with epilepsy subgroups and controls across different activity bands. **Results:** We obtained a modified value for the mean power spectral density in the beta band for the JME group as follows. The connectivity analysis showed that, in general, there was increased linear connectivity in the DMN for the JAE, JME, and EGCTSA groups compared to the healthy controls. Reduced linear connectivity between regions of the DMN was found for DR-IGE. **Conclusions:** Spectral analysis of electroencephalography (EEG) for generalized epilepsy syndromes seems to be less informative than connectivity analysis for DMN. DMN connectivity analysis, especially for DR-IGE, opens up the possibility of finding biomarkers related to drug response in IGE.

## 1. Introduction

According to the International League Against Epilepsy (ILAE), epilepsy is a brain disorder generally defined as follows: 1—at least two unprovoked (or reflex) seizures occurring >24 h apart, 2—one unprovoked (or reflex) seizure and a probability of further seizures similar to the general recurrence risk (at least 60%) after two unprovoked seizures occurring over the next 10 years, and 3—diagnosis of epilepsy syndromes [1].

Three idiopathic generalized epilepsies (IGEs) that are generalized epilepsy syndromes with polygenic causes are juvenile absence epilepsy (JAE), juvenile myoclonic epilepsy (JME), and epilepsy with generalized tonic–clonic seizures alone (EGTCSA) [2].

Patients with JAE experience both absence seizures and generalized tonic–clonic seizures. Compared to childhood absence epilepsy, JAE has fewer (less than daily) absence seizures and seizures are associated with less loss of awareness. A seizure is also triggered by drowsiness, and the duration ranges from 5 to 30 s, during which patients can usually respond to some commands [3].

A common epilepsy syndrome affecting adolescents around puberty, juvenile myoclonic epilepsy, involves bilateral myoclonia without loss of consciousness, generalized tonic–clonic seizures, and infrequent absence seizures. Typically, seizures occur shortly after awakening and are precipitated by sleep deprivation, as Janz and Christian described in 1957 [4].

The term “epilepsy with grand mal seizures on awakening” was used in the past to describe epilepsy with tonic–clonic seizures alone (EGTCSA). Generalized tonic–clonic seizures of variable frequency usually begin in the second or early third decade of life and are typically triggered by sleep deprivation [5].

Although these epileptic syndromes are traditionally known to have a good prognosis, there are situations of drug resistance that require comprehensive approaches. There is the idea that these epilepsies are not really benign. They are a lifelong pathology associated with various comorbidities. However, pharmacoresistance in IGEs remains a controversial issue, as the authors emphasize the importance of distinguishing between true resistance and pseudo-resistance [6].

The resting functional connectivity of IGEs has been extensively studied over the last two decades. Recent research pointed out that functional connectivity analysis and network analysis can be used to diagnose epilepsy and guide patient-specific therapies. The cognitive and behavioral comorbidity of IGEs has motivated research into the connectivity of the brain at rest [7].

The default mode network (DMN) is a resting state network that integrates information from primary function and cognition networks. Studies have detected functional connectivity changes in this network in patients with IGEs (with generalized tonic–clonic seizures) and indicated that these patients can be discriminated from healthy controls [8]. There is also evidence that delta oscillations are significantly different in IGE patients with electrical discharges on the EEG at rest compared to healthy controls in the medial frontal gyrus (BA 9). As a result, patients with IGEs may have different DMN local activity compared to healthy individuals [9].

In a recent, high-density EEG study involving 20 patients with genetic generalized epilepsy and 20 controls, functional connectivity and the hub disruption index were investigated. Compared to controls, patients showed increased connectivity in prefrontal regions and default mode networks but no influence on whole-brain network topology. There seems to be a potential use for this topological reorganization as a diagnostic tool, according to the authors [10].

In studies using EEG-fMRI in patients with IGEs, it has been suggested that DMN connectivity could be used as a biomarker for drug resistance. The researchers found that valproate resistance and uncontrolled seizures significantly reduced DMN connectivity compared to epilepsy alone [11].

A more comprehensive view is suggested by combining power spectrum density and network connectivity studies. However, the existence of a large number of functional connectivity parameters requires a careful approach to establish correlations between them and the power spectral density. Association levels may be affected by the functional connectivity method used to estimate patterns of interactions [12].

The idea that an interictal EEG can be used for diagnosing appeared in the previous decade. We mention here a study with IGE and healthy controls that utilized a computer model of dynamic networks, which derived the network based on the degree of synchrony between EEG channels and the normalized power spectrum of the clinical data. Based on the findings of this study, it was concluded that this biomarker could readily assist in diagnostics [13].

The aim of our study was to determine if there were differences in the mean value of power spectral density between patients with JAE, JME, and EGTCSA and healthy controls in different activity bands. Additionally, we tried to identify differences in linear connectivity among these patients. The study also attempted to identify particularities among patients with drug-resistant IGE (DR-IGE).

There is a parallel between power spectral density analysis and lagged linear connectivity between different groups of patients with generalized epilepsy that could offer new perspectives for the development of biomarkers in drug-resistant epilepsy. There will be an argument in favor of one of the two methods of analysis in generalized epilepsies according to the results.

## 2. Materials and Methods

Participants

The present study included 39 right-handed patients with IGE who underwent an EEG in our unit (Elytis Hospital Hope, Iasi, Romania) in the last six years and 39 age-matched, healthy controls. Of these, 23% had JME, 18% had JAE, and 59% had EGTCSA. Another group of 12 patients with drug-resistant IGE (DR-IGE) and 12 healthy controls were included in the second part of this study.

In this retrospective, case–control study, EEG and clinical data were analyzed for every group study. We included both males and females who fulfilled the criteria of having a generalized epileptic syndrome, with or without medication at the time of the EEG recording, aged between 18 and 60 years. Patients with a history of stroke, tumor, trauma, or abnormal MRIs and patients with concomitant structural epilepsy were excluded.

For the drug-resistant group, the clinical data for each patient were reviewed in accordance with the criteria currently in use. Control subjects were healthy people at the time of the EEG recording who had a normal electroencephalogram in the hospital’s database.

This study was conducted in accordance with the tenets of the Helsinki Declaration and received institutional and ethical approval. It was approved by the University of Medicine and Pharmacy “Grigore T. Popa” Iasi Ethical Committee (approval no. 381/17 January 2024) and Elytis Hospital Hope Ethical Committee (approval no. 2188/12 December 2023).

EEG recording

The EEG was recorded in a soundproofed, normally lit room, using a 19-scalp electrode cap (Cadwell Industries, Inc., Kennewick, WA, USA). The sampling rate frequency was 256 Hz and the impedance was below 5 kΩ.

A certified neurologist examined the typical EEG discharges and reviewed the electroclinical criteria for each epileptic syndrome. The selected EEG epochs were between discharges, according to the criteria listed below: (a) presence of posterior alpha rhythm; (b) absence of drowsiness/sleep EEG features; (c) absence/fewest artifacts (blinking, pulse, sweating, electrode artifacts, etc.); (d) absence of ictal/interictal epileptic discharges (according to ILAE criteria); (e) ≥5 s distance from hyperventilation or photic stimulation. Recordings lasting less than 20 min, recordings with too many artifacts on visual examination, and recordings with focal slowing were excluded.

The drug-resistant group met the same electrophysiological criteria, including those with typical ictal/interictal discharges and those without changes to their EEGs, but also met the pharmacoresistance criteria.

EEG preprocessing

Once the EEG had been selected, we went through a series of steps to process the signals we had obtained in v2020 MATLAB R2023b (MathWorks, Inc., Natick, MA, USA), EEGLAB toolbox. The following steps were performed: (a) a further session of artifact removal; (b) investigation with high- and low-pass filters at 0.5 and 40 Hz; (c) re-referencing of the data for the average reference; (d) interpolation of channels with bad signals; (e) selection of 60 epochs of 2 s each for each patient. Analyzing the independent components was the next step. After checking the activity power spectrum for each component and detecting extra-brain and brain components, we removed a maximum of 3 independent components for each patient—those that contained the highest percentage of artifactual components. The selected date was exported into LORETA (low-resolution brain electromagnetic tomography) using the plugin Loreta 2.0.

Spectral analysis of EEG

This analysis was performed using the Signal Analyzer tool in the MATLAB workspace. We determined the mean power spectral density (log-transformed) in each band defined using a specific algorithm in MATLAB for every subgroup (patients with JAE/JME/EGTCSA/drug-resistant IGE) and the corresponding control subgroup. The band frequencies defined for brain waves were those commonly used: alpha (9–12 Hz), beta (13–30 Hz), theta (4–8 Hz), delta (1–3 Hz). The results represent an average for log power spectral density 10*log⁡10 in every band described. In selecting this parameter, we took into account the preprocessing steps used before the analysis.

LORETA

It is possible to calculate coherence between time series in LORETA (low = resolution brain electromagnetic tomography) by recording scalp electric potential differences extracranially. Studying brain interconnectivity requires a time series of electric neuronal activity [14].

Using the LORETA inverse solution (from EEG/MEG measurements), the electric neuronal activity distribution in three dimensions had the maximum degree of synchronization, in terms of orientation and strength, between neighboring neuronal populations (adjacent voxels). LORETA has been validated by several studies (20 papers from 2001 to 2002) [15].

**Figure 1:**
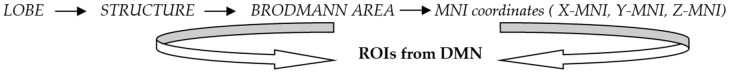
Steps in selecting regions of interest from the default mode network.

We defined in LORETA twenty ROIs (regions of interest) from the default mode network using MNI (Montreal Neurological Institute) coordinates. In this way, we created ROIs from brain regions belonging to the default mode network: BA 10 (middle frontal gyrus), BA9 (medial frontal gyrus), BA8 (medial frontal gyrus), BA 21 (middle temporal gyrus), BA 36 (fusiform gyrus), BA 23 (posterior cingulate), BA 32 (anterior cingulate), BA 30 (parahippocampal gyrus), BA 39, 40 (parietal inferior lobe) [16]. In Figure 1, there is a schematic figure of ROI selection.

Linear connectivity between these regions of interest was performed for every frequency band (σ, θ, α1, α2, β1, β2, β3, Ω) in every patient. This was followed by the statistics for each of the subgroups of patients and control patients of the same age.

Statistical Analysis

Descriptive statistics and χ^2^ tests were performed in Excel, version 2403 (Microsoft Corporation, Redmond, WA, USA). The analysis for power spectral density was performed in GraphPad Prism 10.2.3 (403) using paired t-tests, two-tailed, with a 95% level of confidence for every epilepsy subtype (JAE, JME, EGTCSA, DR-IGE) and healthy controls. The level of significance was *p* < 0.05.

As for the connectivity analysis, this was performed using the statistics of the LORETA program (to measure dependence or obtain time-varying spectra for paired samples). Data from electroencephalograms processed in MATLAB EEGLAB were previously exported in LORETA. The following steps were performed in LORETA’s own statistics program. In LORETA, a nonlinear method was used to find similarities in the frequency domain (a lagged component was obtained that had only physiological components based on normalized Fourier transforms). There were measures of “similarities” between two multivariate time series. In order to estimate functional connectivity accurately, artifacts were removed and physiological, lagged measures were taken [17].

We used a *t*-test for paired groups (A = B) with a variance smoothing parameter of 0 and 5.000 randomizations for multi-comparison correction. After these tests, threshold values were obtained (“time-varying log spectra”) and a file was generated with extremes of probability (ExtremePs or *p*-value), the corresponding maximal thresholds, and thresholds at values of *p* < 0.01, *p* < 0.05, and *p* < 0.10, with *p* < 0.05 for statistical significance. We used the values corresponding to ExtremePs < 0.05 in the “connectivity viewer” to visualize the connectivity of regions of interest that had changes from a statistical point of view. In LORETA, it was established if there was a difference between generalized epilepsy patients and healthy controls regarding “lagged linear connectivity”.

## 3. Results

We found no differences in mean PSD between the control group and the JAE group in the alpha (9–12 Hz), beta (13–30 Hz), theta (4–8 Hz), and delta (1–3 Hz) bands.

In the connectivity analysis, we obtained the values summarized in Table 1A.

**Figure 2:**
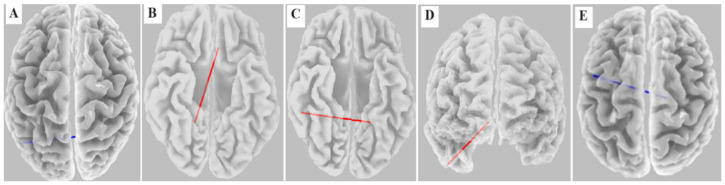
Linear connectivity obtained in LORETA for (**A**) superior view, (**B**) inferior view—JAE, (**C**) inferior view—JME, (**D**) anterior view—EGTCSA, and (**E**) superior view—DR-IGE. Blue = decreased; red = increased.

For ExtremeP = 0.04620 (*p <* 0.05), we obtained a negative value (−5.849) that corresponded to lower linear connectivity (blue in Figure 2A) between the fusiform gyrus, BA 36 from the left temporal lobe and the right posterior cingulate gyrus, BA 23 from the limbic lobe in the delta band. For ExtremeP = 0.00640 (*p <* 0.05), the corresponding value of 5.806 was for increased linear connectivity (red in Figure 2B) between the left anterior cingulate, BA 32 and the right posterior cingulate gyrus, BA 23 in the α1 band.

In the JME group, we found in the paired T-test (two-tailed) a *p*-value of 0.0266 (*p <* 0.05) for mean PSD in the beta band. As a result, the mean PSD in the beta band in the JME group was statistically different from the mean PSD in the control groups.

In Table 1B are the results of the connectivity analysis in the JME group.

ExtremeP = 0.01580 (*p <* 0.05) corresponded to a value of 5.254, indicating higher linear connectivity between the left posterior cingulate gyrus, BA 23 and the right fusiform gyrus, BA 36 in the delta band (Figure 2C).

The EGTCA group has no difference in mean PSD compared to the healthy control group. Regarding the connectivity analysis, for ExtremeP = 0.02320 (*p <* 0.05), a value of 1.597 was found (Table 1C). For this value, higher linear connectivity between the right anterior cingulate, BA 32 and the right middle temporal gyrus, BA 21 in the delta band was found (Figure 2D).

In DR-IGE, we did not find any statistical difference regarding mean PSD. The connectivity analysis revealed ExtremeP = 0.01280 (*p <* 0.05), with a corresponding negative value (−6.100) (Table 1D). This result indicated lower linear connectivity between the left middle frontal gyrus, BA 8 and the right fusiform gyrus, BA 36 (Figure 2E).

**Figure 3:**
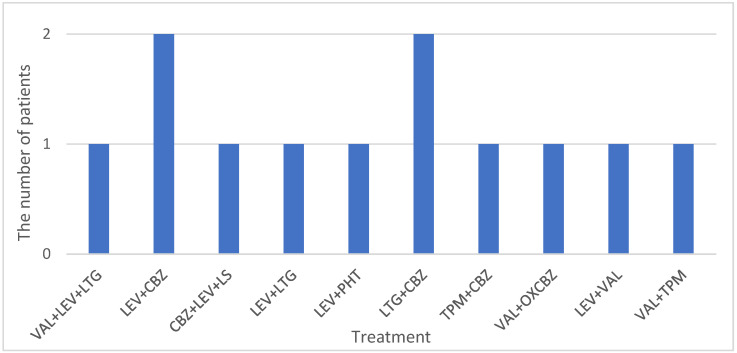
Treatment for DR-IGE group. Abbreviations: LEV = levetiracetam, VAL = sodium valproate, LTG = lamotrigine, CBZ = carbamazepine, TPM = topiramate, PHT = phenytoin, OXCBZ = oxcarbazepine, LS = lacosamide.

For the DR-IGE group, the shortest duration since seizures began was 6 years and the longest was 32 years. The mean age for this group was 30.66 ± 9.25 (median 32.5 years old). A statistical difference was not detected between the DR-IGE and controls regarding age and sex distribution, with *p* = 0.6857 according to the χ2 test. Figure 3 shows the treatment for DR-IGE at the time of EEG recording.

## 4. Discussion

Studies have shown a correlation between higher IGE spectral power and greater neuronal synchrony. In addition, an increase in power was observed before myoclonic seizures, which supports the idea that this is related to seizure susceptibility [18]. Our results concerning the power spectrum density in the beta band for JME could be related to myoclonia susceptibility, particularly given the fact that patients rarely mentioned it. Other groups did not have any modifications regarding this parameter. The lagged linear connectivity analysis provided much more relevant results in this regard.

In recent years, functional connectivity studies have increased, identifying particularities of the default mode network in generalized epilepsy patients, but their causality remains unclear. The role of medication, the duration of the disease, and additional cognitive problems remain to be determined.

Several explanations for impaired default mode network (DMN) functional connectivity in patients with generalized epilepsy have been proposed. This may reflect chronic dysfunction of the functional architecture due to neurophysiological dysfunction or intermittent perturbations disrupting cortical networks [19].

Using resting-state functional magnetic imaging to examine dynamic functional connectivity in JAE, it was found that nodes from DMN were changed in striatal–cortical networks. The connection between the dorsal caudate putamen and the precuneus from the DMN seemed excessively stable. In this study, striatal–subcortical networks were found to be more stable and striatal–executive circuits were found to be more variable in JAE [20].

A synchronization between anterior and posterior regions in the alpha band in DMN could play a role in ictogenesis, as indicated in our study by elevated linear connectivity between the left anterior cingulate and the right posterior cingulate.

A fMRI study on drug-naïve JME and healthy controls revealed dysconnectivity in DMN. There was an alteration in causal influence flows between the medial prefrontal cortex and angular gyrus in a state-specific manner, which was related to disease severity. The findings are related to the neuropsychological processes involved in JME [21]. We found increased connectivity between the left posterior cingulate gyrus and the right fusiform gyrus in the JME group, and we attribute this finding to the susceptibility to myoclonia (as mentioned for PSD in the beta band).

A study that utilized graph theory for the topological analysis of functional networks found an increase in inter-network connectivity in the DMN of children with generalized tonic–clonic seizures compared with healthy controls [22].

In our study, the linear connectivity between the right anterior cingulate and right middle temporal gyrus was higher in the EGTCSA group.

Using stereo-EEG in patients with focal drug-resistant epilepsy, a functional and effective connectivity study found that the anterior cingulate cortex connected with the mesial temporal, orbitofrontal, and prefrontal cortex [23]. Among our IGE subgroups, we found alterations in connectivity between the cingulate cortex and other regions of interest from the DMN, underlining the importance of this region in generalized epilepsy.

Genetic generalized epilepsies are associated with structural and functional connectivity alterations. A hub reorganization was found in these patients and all genetic generalized epilepsy syndromes have hubs in the areas of DMN as follows: superior parietal and posterior cingulate cortex in absence epilepsy; middle frontal cortex, posterior cingulate cortex, precuneus, and parahippocampal cortex in JME, superior parietal cortex, caudal cortex, anterior cingulate cortex, and temporal pole cortex in EGTCSA [24].

Brain network analysis is emerging as a potential biomarker for idiopathic generalized epilepsies (IGEs). IGEs exhibited functional hyperconnectivity, as determined by a systematic review of 22 studies assessing functional connectivity and networks through magnetoencephalography and EEG. The changes were observed on a normal background, without epileptiform discharges, which is considered normal in clinical evaluation. This was found in the review, while the meta-analysis did not find any changes [7].

Drug resistance in IGE is controversial in terms of treatment and mechanism. Functional MRI at rest in 10 patients with well-controlled IGE, 23 drug-resistant IGE patients, and 34 healthy controls showed that the topology of the interictal network of IGE was more regular and had higher global connectivity compared to the controls. These findings were associated with a susceptibility to transition to seizures. They did not find any differences that were influenced by seizure control. However, the authors pointed out the small sample size of the study and the difficulties in classifying the response to antiepileptic drugs [25].

According to a study using EEG-fMRI to quantify DMN connectivity in 60 epilepsy patients and 38 healthy controls, valproate resistance and uncontrolled seizures were associated with a greater reduction in DMN connectivity compared to epilepsy alone [11].

Other studies have also highlighted the significance of the DMN among resting-state networks in identifying IGE patients from controls [8,26,27,28].

It was found in our DR-IGE group that connectivity between the left middle frontal gyrus and the right fusiform gyrus was reduced, which is consistent with the literature suggesting reduced connectivity within the default mode network in this group.

Connectomics could be useful in selecting patients for thalamic DBS (deep brain stimulation) for drug-resistant epilepsy based on the connectome of the targeted nucleus with epileptogenic foci. Researchers found that patients with drug-resistant epilepsy who are not candidates for resective surgery responded well to CMN DBS (centromedian nucleus of the thalamus DBS) for primary generalized tonic–clonic seizures with multiple epileptic foci in the precentral motor and primary somatosensory cortex [29].

A retrospective analysis of six epilepsy patients treated with ANT DBS (anterior nucleus of the thalamus DBS) found increased DMN connectivity in responders in the following regions: posterior cingulate cortex, medial prefrontal cortex, inferior parietal lobule, and precuneus [30].

It might be possible to use interindividual differences in DMN functional connectivity as biomarkers in selecting patients, determining stimulation parameters, and optimizing neuromodulation procedures [31]. There was a positive correlation between improvement after CM-DBS in generalized drug-resistant epilepsy and the connection between the centromedian nucleus of the thalamus and the brainstem, cerebellum, and sensory-motor cortices. More research on brain network characteristics will enhance the identification of optimal targets for stimulation in epilepsy syndromes [32].

It was suggested that fMRI connectivity could be used to predict outcomes after surgery in patients with intractable epilepsy [33]. The results obtained from analyzing the connectivity to the group with pharmacoresistance can be very useful and can be used as an entry point to determine if it is possible to use this to select patients most likely to benefit from invasive treatments.

In a study that involved 42 children with focal cortical dysplasia pharmacoresistant epilepsy and 116 controls, the functional connectivity analysis of resting-state MRI showed diminished functional connectivity in the group with epilepsy, independent of the cortical dysplasia location. Researchers analyzed dorsal attention, default mode, and control functional networks, finding diminished functional connectivity in these networks and proposing these patterns as potential neuromarkers [34].

In order to find valid biomarkers for epilepsy surgery outcomes, further research is needed in this area, especially multicentric studies. A DMN connectivity study before and after invasive treatment could identify biomarkers of outcomes in these patients, and this is an open area of research.

A limitation of the present study is the small number of patients, especially for the drug-resistant group. The small number of electrodes used for electrical source imaging is another source of bias. The lack of subdivision in DR-IGE could be another limitation, but the small number of patients did not allow us to subdivide this group. A limitation of the systematic review itself should be mentioned; there is a need to include more studies on further directions of network connectivity. The results need to be validated and generalized through prospective, multicentric studies.

## 5. Conclusions

There is growing evidence that the default mode network is particularly important in generalized epilepsy. In this study, the DMN connectivity analysis provided a wider perspective than the spectral analysis. In patients with JAE, JME, and EGTCSA, we found increased DMN connectivity between regions of interest, while in patients with drug resistance, we found decreased connectivity. Further studies of DMN connectivity in IGE could identify biomarkers related to drug response. It is possible that this approach will provide new insights into the prognostic factors for epilepsy surgery.

## Figures and Tables

**Table 1:** Statistic results obtained in LORETA for time-varying log spectra for IGE patients.

Epilepsy Type	*t*-Tests	t(0.01)	t(0.05)	t(0.10)	ExtremeP
A. JAE *	One-Tailed (A > B):	6.823	5.806	5.364	0.00640
	One-Tailed (A < B):	−7.118	−5.849	−5.379	0.04620
	Two-Tailed (A < >B):	7.522	6.244	5.731	0.01600
B. JME *	One-Tailed (A > B):	6.117	5.254	4.862	0.01580
	One-Tailed (A < B):	−6.108	−5.224	−4.870	0.05540
	Two-Tailed (A < >B):	6.428	5.586	5.162	0.02940
C. EGTCSa *	One-Tailed (A > B):	1.880	1.597	1.453	0.02320
	One-Tailed (A < B):	−1.881	−1.583	−1.442	0.59780
	Two-Tailed (A < >B):	1.942	1.724	1.589	0.04580
D. DR-IGE *	One-Tailed (A > B):	7.330	5.986	5.519	0.06640
	One-Tailed (A < B):	−7.477	−6.100	−5.596	0.01280
	Two-Tailed (A < >B):	7.954	6.684	5.997	0.02200

* JAE = juvenile absence epilepsy, JME = juvenile myoclonic epilepsy, EGTCSa = epilepsy with generalized tonic–clonic seizures alone, DR-IGE = drug-resistant IGE, ExtremeP = *p*-value, values in red with statistical significance.

## Data Availability

Datasets analyzed in this study are available from the corresponding author upon reasonable request.

## Abbreviations

BA	Brodmann area
DBS	deep brain stimulation
DMN	default mode network
DR-IGE	drug-resistant IGE
EGTCSA	epilepsy with generalized tonic–clonic seizures alone
IGEs	idiopathic generalized epilepsies
JAE	juvenile absence epilepsy
JME	juvenile myoclonic epilepsy
ROIs	regions of interest
